# Lipid droplets meet aging

**DOI:** 10.18632/aging.202883

**Published:** 2021-03-23

**Authors:** Thomas Felder, Florian Geltinger, Mark Rinnerthaler

**Affiliations:** 1 Department of Biosciences, Division of Genetics, University of Salzburg, Salzburg, 5020, Austria; 2 Department of Laboratory Medicine, Paracelsus Medical University, Salzburg, 5020, Austria

**Keywords:** protein homeostasis, aggregates, mitochondria, lipid droplets, cancer

Aging and related diseases are multifactorial processes associated with the intracellular accumulation of damaged proteins and lipids along with their inefficient removal. To preserve cellular homeostasis, cells have developed a broad variety of organelle-specific systems that are highly linked. Removal of misfolded proteins utilizes pathways such as the ubiquitin proteasome system, chaperones, endoplasmic-reticulum-associated protein degradation, asymmetric segregation of proteins, autophagy, several proteases (lysosome/vacuole and mitochondria), the MAGIC (*m*itochondria *a*s *g*uardian *i*n *c*ytosol) system and autophagy (micro-, macro- and chaperone mediated) (for a detailed review see [1]).

**Figure 1:**
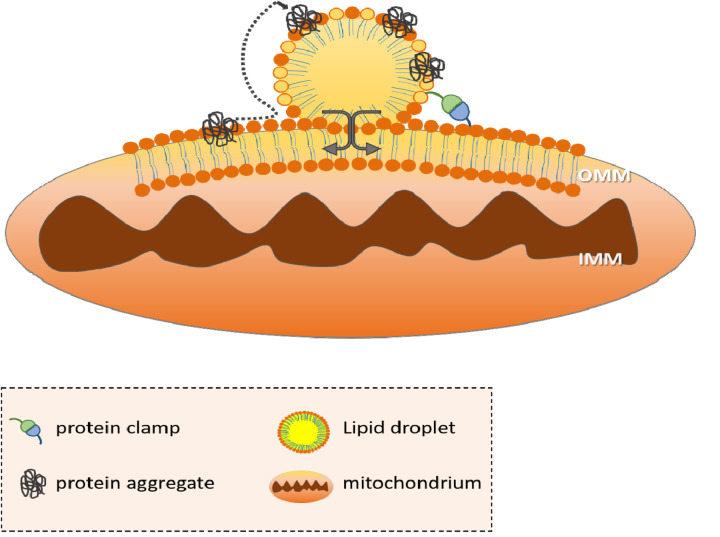
**The mitochondria-LD interaction.** In times of stress, the ER wraps around mitochondria and promotes mitochondrial fission. LDs originate from the ER and thus are in close contact to the OMM. A protein clamp stabilizes the contact between LDs and mitochondria and harmful proteins, protein aggregates as well as mitochondrial lipids (brown head group) are shuttled from mitochondria to LDs. LDs surrounded by a phospholipid monolayer (yellow head group) supply mitochondria with lipids (yellow) for beta-oxidation. OMM (outer mitochondrial membrane); IMM (inner mitochondrial membrane).

Increasing evidence suggests that capacity overload of each pathway may initiate lipid droplet (LD) dependent backup systems. LDs consist of an inner core of neutral lipids such as triacylglycerols and steryl esters, which is coated by a phospholipid monolayer and attached proteins. The main LD function is transient storage of lipids for energy production and membrane biosynthesis along with the prevention of liptoxicity. LDs are highly dynamic organelles and provide an interface for lipid and protein transfer as they physically interact with a pleiotropy of cell organelles such as the ER, lysosomes, peroxisomes and mitochondria. Furthermore, LDs can receive ER resident protein aggregates of damaged and misfolded proteins [2]. Additionally, LDs sequester sterols that act as solvents to dissolve cytosolic protein aggregates [3]. This suggests an important role of LDs as detoxification organelles. Recently, we demonstrated that during stress and aging the number of LDs strongly increases in *S. cerevisae* as well as mammalian cells. Some of these LDs establish close contact sites with the outer mitochondrial membrane (OMM) (Figure 1) [4,5]. Consequently, proteins specifically shuttle from mitochondria to LDs, indicating a trafficking of proteins between these two organelles in *S. cerevisiae* [4]. Among “shuttled” proteins, a strong prevalence for pro- as well as anti-apoptotic molecules [5] became evident. Interestingly, some of the identified proteins (e.g. Ubx2p and Gpa1p) participate in the aging process of yeast cells [4]. Regulation at mitochondria and LDs during aging and stress was not restricted to proteins, but also occurred on the lipidome level. Amounts of phosphatidylinositols and ergosterols (the yeast sterols) increased at mitochondria, whereas levels of these lipids simultaneously decreased at LDs [4], indicative for an exchange process during stress. Finally, these lipid and protein loaded LDs are detoxified in the lysosome/vacuole in a process resembling autophagy, termed microlipophagy [2]. Recent data also indicate that the formation of stable contact sites between LDs and lysosomes can promote a direct transfer of proteins and lipids thus bypassing the necessity of LD degradation as a whole [6]. Ectopic expression of enzymes that increased LD content improved the general fitness of *S. cerevisiae* and reduced the sensitivity for pro-apoptotic stimuli [5]. Furthermore, the accumulation of LDs protected aged cells against stress [7], thereby indicating a potential role of LDs on longevity. Recent findings reported the presence of LDs at the inner side of the nuclear envelope and inside the nucleus of yeast and mammalian cells. These LDs may assist in the regulation of nuclear protein content and remodeling of the nuclear envelope. The involvement in lipid signaling and nuclear receptor function possibly contributes to the transcriptional regulation of ageing and longevity. Although mainly beneficial, LDs can be harmful due to the increase of cellular fitness in transformed cells. LD accumulation occurs in a broad variety of human cancers, in which they increase tumor growth and invasiveness. This adverse effect of LDs is due to their capacity to reduce lipotoxicity and proteotoxicity. Furthermore, LDs provide a constant supply of lipids for tumor cells in energy production as well as membrane biogenesis. All these factors highlight the observation that LDs promote the survival of tumor cells in the presence of chemotherapeutic stress [8]. Therefore, LDs are important mediators of protein homeostasis with a janus-faced character. Increasing the number of LDs is a prerequisite for cellular fitness especially during aging, but in some disease states like cancer this may have a negative impact, which even boosts tumor aggressiveness and reverses the beneficial properties of LDs.
